# Arterial oxygen desaturation during moderate hypoxia hinders sensorimotor performance

**DOI:** 10.1371/journal.pone.0297486

**Published:** 2024-02-23

**Authors:** Jason M. Keeler, Jennifer B. Listman, M. Jo Hite, David J. Heeger, Erica Tourula, Nicholas L. Port, Zachary J. Schlader

**Affiliations:** 1 Department of Kinesiology, H.H. Morris Human Performance Laboratories, School of Public Health-Bloomington, Indiana University, Bloomington, IN, United States of America; 2 Statespace Labs, Inc. New York, NY, United States of America; 3 School of Optometry, Indiana University, Bloomington, IN, United States of America; University Medical Centre Ljubljana (UMCL) / Faculty of Medicine, University Ljubljana (FM,UL), SLOVENIA

## Abstract

**Introduction:**

Moderate hypoxia may impact cognitive and sensorimotor performance prior to self-recognized impairments. Therefore, rapid and objective assessment tools to identify people at risk of impaired function during moderate hypoxia is needed.

**Purpose:**

Test the hypothesis that reductions in arterial oxygen saturation during moderate normobaric hypoxia (F_i_O_2_ = 14%) decreases gamified sensorimotor performance as measured by alterations of motor acuity.

**Methods:**

Following three consecutive days of practice, thirty healthy adults (25 ± 5 y, 10 females) completed three bouts of the tablet-based gamified assessment (Statespace Labs, Inc.) of motor acuity at Baseline and 60 and 90 min after exposure to 13.8 ± 0.2% (hypoxia) and 20.1 ± 0.4% (normoxia) oxygen. The gamified assessment involved moving the tablet to aim and shoot at targets. Both conditions were completed on the same day and were administered in a single-blind, block randomized manner. Performance metrics included shot time and shot variability. Arterial oxyhemoglobin saturation estimated via forehead pulse oximetry (S_p_O_2_). Data were analyzed using linear mixed effects models.

**Results:**

Compared to normoxia (99±1%), S_p_O_2_ was lower (p<0.001) at 60 (89±3%) and 90 (90±2%) min of hypoxia. Shot time was unaffected by decreases in S_p_O_2_ (0.012, p = 0.19). Nor was shot time affected by the interaction between S_p_O_2_ decrease and baseline performance (0.006, p = 0.46). Shot variability was greater (i.e., less precision, worse performance) with decreases in S_p_O_2_ (0.023, p = 0.02) and depended on the interaction between S_p_O_2_ decrease and baseline performance (0.029, p< 0.01).

**Conclusion:**

Decreases in SpO_2_ during moderate hypoxic exposure hinders sensorimotor performance via decreased motor acuity, i.e., greater variability (less precision) with no change in speed with differing decreases in S_p_O_2_. Thus, personnel who are exposed to moderate hypoxia and have greater decreases in S_p_O_2_ exhibit lower motor acuity, i.e., less precise movements even though decision time and movement speed are unaffected.

## Introduction

Military operations require service members to execute a myriad of tasks at or near physiological and/or psychological limits. The successful execution of such tasks requires personnel to utilize a variety of cognitive domains (i.e., executive function, working memory, attention, sensorimotor control, etc.), typically while enduring harsh environmental stressors (e.g., hypoxia, heat, cold, water immersion). Understanding and identifying how each environmental stressor impacts specific cognitive processes is vital to prepare Warfighters for mission success, since there is a limit to an individual’s cognitive capacity [1]. Hypoxia is an environmental stressor that affects all branches of the military. Specifically, with the advent of the airplane, movement of supplies and people can happen faster than ever. However, acute ascent to high altitudes especially in unpressurized aircraft has created the risk of hypoxemia. As humans ascend, the partial pressure of oxygen in the atmosphere drops creating a potentially hazardous environment, in which the arterial oxygen saturation decreases. This results in decreases in oxygen delivery to body tissues that can result in deleterious consequences, like impaired cognitive function, unconsciousness, or death [2–5]. Previous investigations of extreme hypoxic exposure (e.g., F_i_O_2_ ≤ 10% or altitudes over 17,000 ft) have consistently found cognitive impairments, while moderate hypoxic exposures with fractions of inspired oxygen (F_i_O_2_) of 11–14% (equivalent to ~10,000–17,000 ft of altitude) display inconsistencies with either no or slight impairments to cognitive tasks such as executive function, sensorimotor, and working memory [2,6]. Unfortunately, even slight deficits in such complex tasks could lead to unsafe practices and potential mission failure. However, aircrews are consistently exposed to such moderate hypoxic conditions in unpressurized aircrafts, for periods of up to one hour [7]. Thus, tools are needed that can identify detrimental cognitive effects of moderate hypoxia and provide personnel objective feedback, so that safety protocols can be initiated prior to a possible accident.

One potential solution is gamified cognitive assessments, where electronic games can provide objective feedback of subtle alterations in targeted metrics of cognitive function within different domains, specifically complex cognitive domains [8]. Gamified cognitive assessments have a potential benefit of improved intrinsic and extrinsic motivation of the participants, thereby improving adherence and engagement, improving assessment accuracy [8]. The idea of gamifying cognitive assessments has increased in popularity over recent years and is noted as the addition of elements such as scores, leaderboards, badges, and auditory feedback [8–10].

For example, recent investigations into sensorimotor performance have utilized a first-person shooter stylistic game to measure motor acuity [9–11]. Sensorimotor performance is an example of a complex cognitive task that requires the uptake and integration of information from the environment, while maintaining attentional focus to quickly formulate and execute precise motor actions. Motor acuity provides a measure of sensorimotor performance and is defined as the ability to execute actions more precisely and within a shorter amount of time, which may be measured by characterizing the known tradeoff between speed and accuracy (precision) [10]. A gamified task that measures motor acuity could provide valuable feedback to personnel working in hypoxic environments as decrements in sensorimotor performance could inhibit or slow the ability of the personnel to execute job functions or employ necessary safety measures during a crisis. Utilizing gamified cognitive assessments periodically during hypoxic exposure that provide objective feedback could be beneficial to mitigate the risk of poor job performance and possible workplace accidents. The effect of moderate hypoxic exposure on sensorimotor performance, and specifically on motor acuity, has not been tested previously.

The primary purpose of this investigation was to evaluate whether motor acuity in a new tablet-based gamified sensorimotor task (Adaptive Reflexshot; Statespace Labs, Inc.) is sensitive to moderate hypoxia. To account for individual differences in arterial oxygen saturation to a given F_i_O_2_ [5], this investigation aimed to take into account the magnitude of hypoxemia on sensorimotor performance. Therefore, we tested the hypothesis that motor acuity is hindered by reductions in arterial oxygen saturation during moderate normobaric hypoxia (F_i_O_2_ = 14%).

## Methods

### Experimental design

Before any study activities commenced, this study was approved by the Institutional Review Board at Indiana University (#12623) and was subsequently performed in accordance with the standards set by the latest revision of the Declaration of Helsinki, except for the registration in a database. Participants provided written consent prior to taking part in this study. This study utilized a single blind, block randomized, balanced, cross-over design. Participants were recruited for this study from June 2022 until November 2022.

**Fig 1:**
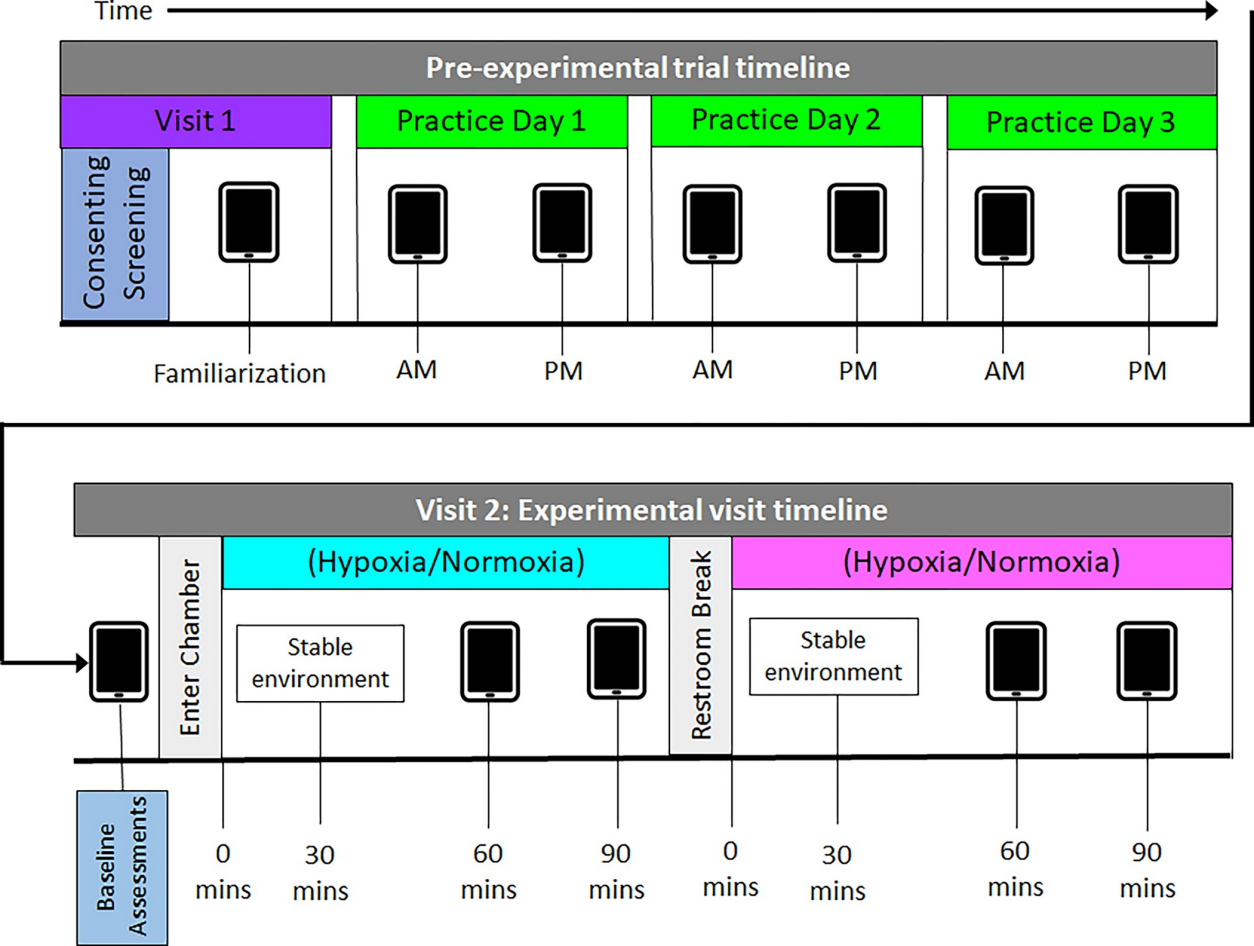
Study timeline. Session 1 included consenting, screening, and familiarization. Familiarization of the gamified sensorimotor task consisted of a demonstration of the task followed by 3 sets of the gamified sensorimotor task. Following Session 1, participants completed two practice sessions per day of the gamified sensorimotor task for the three consecutive days prior to returning to the lab for the experimental session. Each practice session consisted of playing 3 sets of the gamified sensorimotor task, and participants were asked to complete one practice session in the morning and the other in the afternoon/evening with at least four hours between practice sessions. Session 2 included baseline measures of body mass, heart rate, oxygen saturation, and the baseline assessment (comprising 3 sets of the task). After completing the baseline assessment, participants sat in a chair in the environmental chamber and were allowed to watch a documentary of their choosing between assessments. The environmental chamber was then set to a hypoxic (F_i_O_2_ = 14%) or normoxic (F_i_O_2_ = 21%) environment (counterbalanced across participants). Participants were blinded to order of exposure. Participants completed an assessment (3 sets of the task) after 60 min of exposure to either hypoxia or normoxia and again after 90 min of continuous exposure, to see if longer exposure impacts cognitive performance. Following the 90 min assessment, participants were given a 5 min bathroom break and then reentered the chamber. The F_i_O_2_ in the environmental chamber was changed to the opposite environmental condition. Again, participants completed an assessment after 60 min of exposure to the experimental conditions and again after 90 min of exposure. Data were upload to a server and participants were free to leave the lab after deinstrumentation.

Participants reported to the laboratory on two separate occasions, with at least 72 hours between visits, and within 48 hours after the final practice day (Fig 1). Session 1 included consenting, screening, and familiarization. Session 2 was the experimental session. Familiarization of the gamified sensorimotor task during Session 1 consisted of a demonstration of the task followed by 3 sets of the gamified sensorimotor task. After each set, the study personnel answered any questions. Following Session 1, participants completed two practice sessions per day of the gamified sensorimotor task for the three consecutive days prior to returning to the lab for Session 2, the experimental session. Each practice session consisted of playing 3 sets of the gamified sensorimotor task, and participants were asked to complete one practice session in the morning and the other in the afternoon/evening with at least four hours between practice sessions. The familiarization and practice sessions were incorporated as previous work has demonstrated a learning plateau following four practice sessions [9,11,12], thereby minimizing learning during the experimental session.

### Participants

An a priori power analysis was carried out with G-Power version 3.1.9.4 software. We utilized data from our previous investigation of hypoxia and its effects on a first person shooter stylistic gamified sensorimotor test (Gridshot, [11]). Thus, utilizing this previous work to estimate sample size for the current study, a Cohen’s d_z_ effect size of 0.78 was found when looking at Gridshot shot precision during hypoxic exposure. Using this effect size and assuming a moderate positive correlation among repeated measures (r = 0.5), a sample size of at least 23 participants was needed to detect a significant interaction (condition x time) using standard parameters of 1-β = 0.80 and α = 0.05. However, due to the exploratory nature of this study we increased our sample size requirements to 30 to improve the identification of differences between the conditions. Therefore, thirty healthy adults (26 ± 5y, 10 Females) were recruited and completed the study. Participant characteristics were height: 176 ± 9 cm, weight: 77 ± 16 kg, and body mass index: 25.1 ± 3.9 kg∙m^-2^. Participants self-reported being moderately active, nontobacco users, and free from any psychological, cardiovascular, and pulmonary diseases. Participants completed the Montreal Cognitive Assessment (MoCA) version 8.1 and had to score the age group median score of at least of 26 out of 30 to participate in the study (average score: 29 ± 1)[13]. Participants self-reported to be consistent video game players with an average of 1.6 ± 1.4 h of video game play per week. Participants were also free of sickle cell trait and disease. Females self-reported to be normally menstruating and were confirmed not to be pregnant via pregnancy test. Due to the within subject design and experimental trials occurring on the same day, females were tested across the menstrual cycle.

### Physiological instrumentation and measurements

Body height (cm) and mass (kg) were measured using a stadiometer (Holtain Limited, Seritex, Wales, UK) and scale (Sauter, Balingen, Germany). Heart rate (bpm) and an estimate of arterial oxygen saturation (S_p_O_2_, %) were measured continually via a pulse oximetry sensor (Nellcor N600x, Medtronic Inc., USA) placed on forehead. Blood pressure was measured using an automated blood pressure cuff (Suntech CT40, SunTech Medical, USA). Participants were also asked to identify the environment (normoxia or hypoxia) they were in at 60 and 90 min of exposure via questionnaire.

### Gamified sensorimotor task

**Fig 2:**
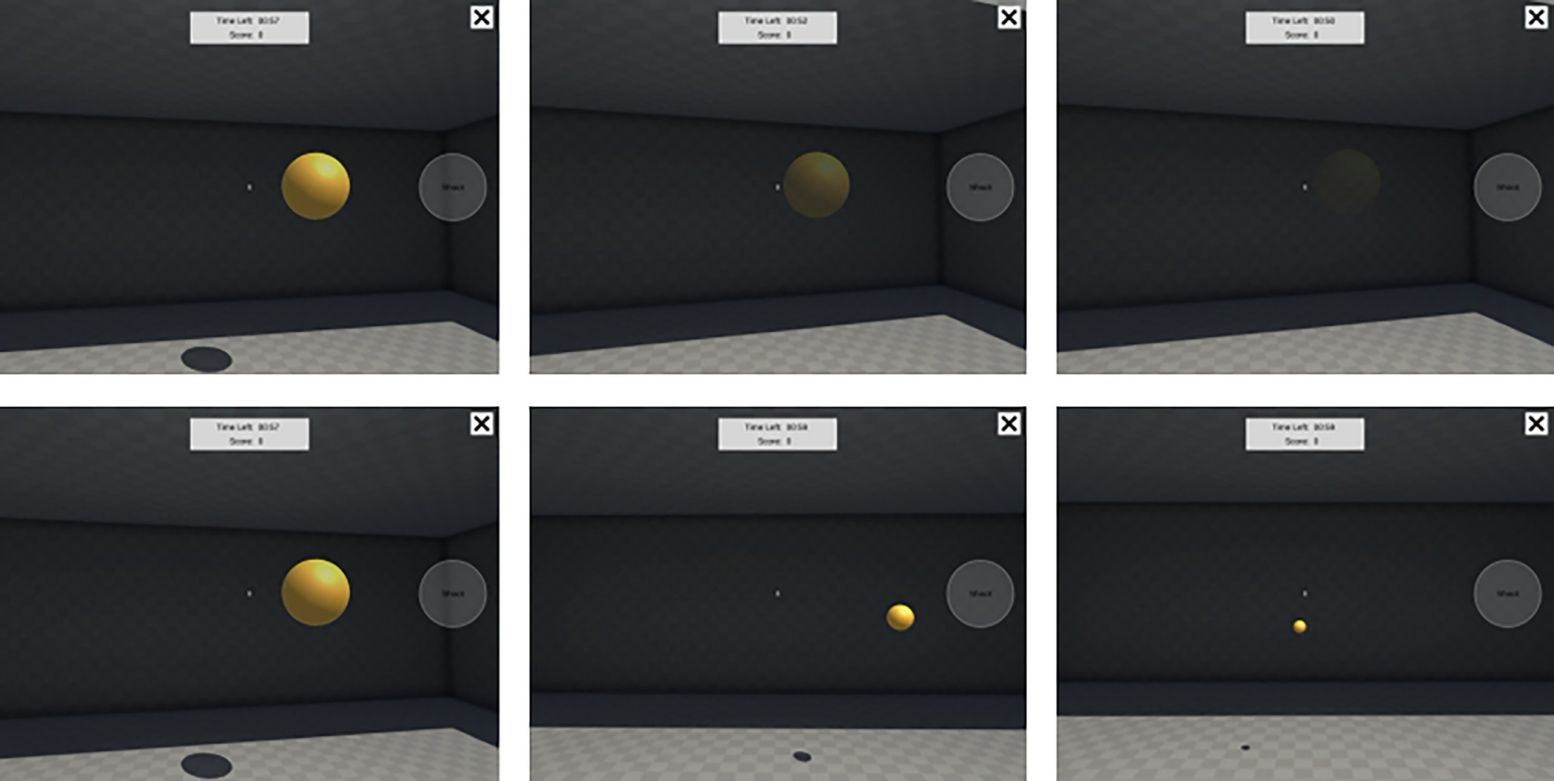
Adaptive Reflexshot. Participants had a limited time to destroy each target before it “timed-out” and disappeared. If the target timed-out, no points were awarded to the player for that target. To provide a visual cue of time remaining, each target gradually became more transparent and then disappeared. The three panels in the top row of Fig 2. shows a time series of screenshots as the target becomes progressively more transparent. Three different target sizes (large, medium, small) were presented in different rounds of the task, and target duration is titrated separately for each target size. The three panels in the bottom row of Fig 2 show examples of three different target types or sizes.

Participants completed the gamified sensorimotor task (Adaptive Reflexshot) via a tablet (iPad, Apple Inc.) loaded with the Brain Lab prototype application developed by Statespace Labs Inc., New York, NY, USA. Adaptive Reflexshot is a first person shooter stylistic sensorimotor assessment task designed to measure motor acuity by characterizing the tradeoff between speed and precision [10]. The Adaptive Reflexshot task involved moving the iPad to aim and shoot at a series of targets. All rounds of Adaptive Reflexshot were completed in the upright, standing position, as orthostasis provides a greater circulatory challenge [14]. Participants held the tablet out in front of the body with a hand on either side and then tapped a button on the tablet screen with their thumb to shoot at the targets. To aim at each target, participants had to move the whole tablet using the gyroscopic technology to align the crosshairs on each target. Each round of play was 1 min in duration. During each round, a series of targets were presented, one at a time, in a randomized location confined to an elliptical region in front of the player’s virtual avatar. Participants were provided immediate feedback after each shot was fired via an audible positive or negative sound. Participants had a limited time to destroy each target before it “timed-out” and disappeared. If the target timed-out, no points were awarded to the player for that target. To provide a visual cue of time remaining, each target gradually became more transparent and then disappeared. This motivated the players to be as fast as possible. The three panels in the top row of Fig 2 shows a time series of screenshots as the target becomes progressively more transparent. The target presentation duration was titrated according to performance: the duration was decreased (it became transparent more quickly) on the next trial after a target was destroyed and the duration was increased (it became transparent more slowly) on the next trial after a target timed-out. Three different target sizes (large, medium, small) were presented in different rounds of the task, and target duration is titrated separately for each target size. The three panels in the bottom row of Fig 2 show examples of three different target types or sizes.

Each sensorimotor assessment comprised 3 sets of the Adaptive Reflexshot task, and each set comprised 3 rounds, one for each of the 3 target sizes. The order of target size rounds within a set was counter-balanced across participants. Throughout each round of play, a composite score was visible to participants at the top of the tablet screen. A final score was displayed after the end of each round. The scoring was designed to incentivize players to prioritize precision over speed when targets were small, and to prioritize speed over precision when the targets were large. Points were added to the score when targets were hit and points were subtracted from the score for shots that missed the target. The number of points added for each target destroyed depended on both target size and duration such that small, briefly presented targets had the greatest value and large, long duration targets had the least value. The scoring method was developed, based on a different group of study participants, to be proportional to the cumulative miss rate across a population. The number of points subtracted for each missed shot was 1/5^th^ the number of points added for hitting that target.

Performance in the Adaptive Reflexshot task was analyzed to measure two key performance metrics, separately for each target size: 1) shot time (median elapsed time between target appearance and the first shot fired at that target, in units of seconds), and 2) shot variability (median angular distance between target center and the location of the first shot, in units of degrees). The median angular distance, the spread of the distribution of shots around the target centers, is a measure of variability. We used the median instead of the mean because median is robust to outliers.

**Fig 3:**
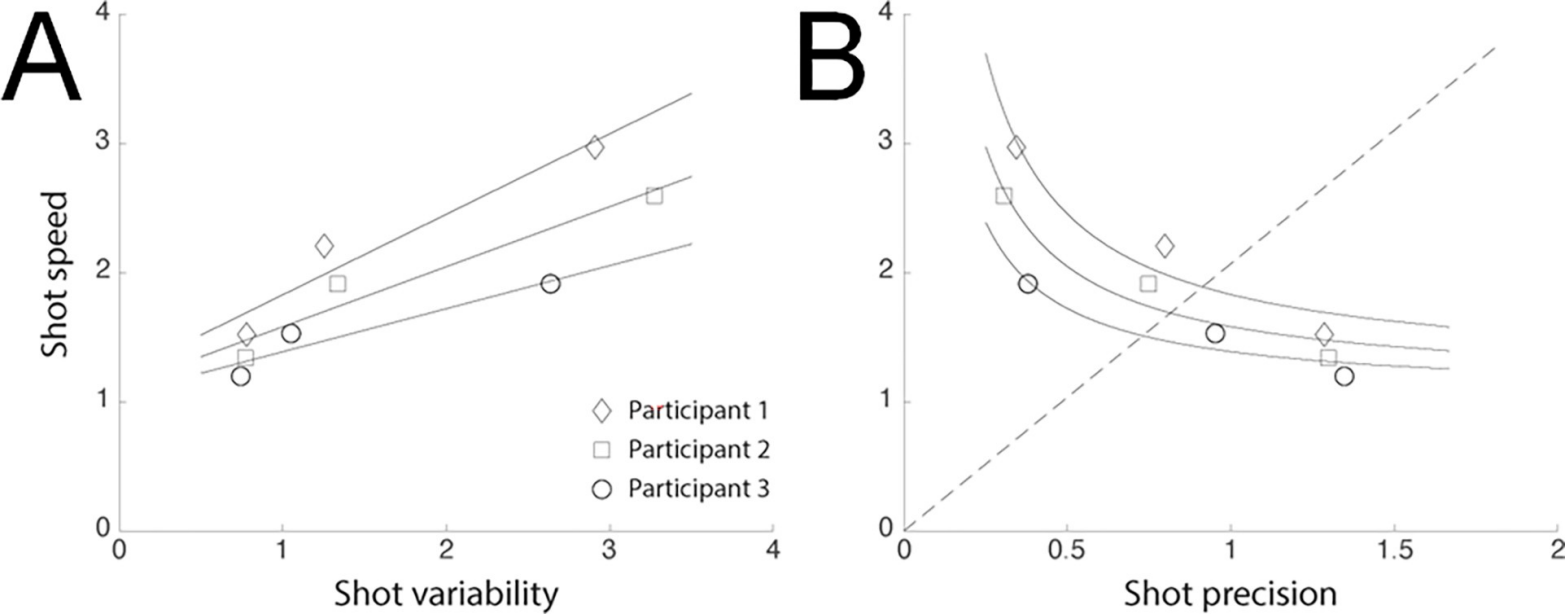
Tradeoff between speed and precision. Fig 3 illustrates the tradeoff between speed and precision for three example participants. Fig 3A plots shot speed (1/(shot time)) versus shot variability. Fig 3B plots shot speed (1/(shot-time)) versus shot precision (1/(shot-variability)). For each participant, the leftmost plot symbol in Fig 3A (rightmost in Fig 3B) corresponds to the small target, the rightmost plot symbol in Fig 3A (leftmost in Fig 3B) corresponds to the large target, and the plot symbol in the middle corresponds to the medium target. The solid lines in Fig 3A are best-fit lines to the data from each participant. The curves in Fig 3B represent the same best fits, replotted by replacing precision (1/variability) for variability.

Motor acuity may be assessed in terms of the tradeoff between speed and precision (Fig 3). Human performance in almost any task is known to exhibit tradeoffs between speed and precision (often called a speed-accuracy tradeoff, although “precision”, not “accuracy”, is being measured). The different target sizes and target-presentation durations in the Adaptive Reflexshot task incentivized players to either make faster and less precise shots, or slower and more precise shots. Fig 3 illustrates the tradeoff between speed and precision for three example participants. Fig 3A plots shot speed (1/(shot time)) versus shot variability. Fig 3B plots shot speed (1/(shot-time)) versus shot precision (1/(shot-variability)). For each participant, the leftmost plot symbol in Fig 3A (rightmost in Fig 3B) corresponds to the small target, the rightmost plot symbol in Fig 3A (leftmost in Fig 3B) corresponds to the large target, and the plot symbol in the middle corresponds to the medium target. The solid lines in Fig 3A are best-fit lines to the data from each participant. The curves in Fig 3B represent the same best fits, replotted by replacing precision (1/variability) for variability. As expected, participants were faster but more variable (less precise) for large targets, and slower but less variable (more precise) for small targets, following the task incentives. Of the three participants shown in Fig 3, the participant represented by the diamond-shaped plot symbols had the best motor acuity (the curve in Fig 3B is further up and to the right, i.e., fastest and most precise) and the participant represented by the circular plot symbols had the worst motor acuity.

It was critical to utilize multiple target sizes and to measure both speed and variability (or, equivalently, precision), as it is not possible to use a single performance metric as a proxy for motor acuity [10]. For instance, measuring only shot speed for only one target size can be confounded by participants simply shooting faster with no penalty for being inaccurate and imprecise, a strategy that does not reflect a high level of skill. Even measuring both shot speed and shot precision, but for only a single target size, would provide only 1 data point on the speed-precision tradeoff curve. Consequently, there would be no way to determine if a change in performance under different conditions was due to a shift along the same tradeoff curve (i.e., same motor acuity but different priority for speed vs precision) versus a change in motor acuity (i.e., a shift to a different speed-precision tradeoff curve).

### Visit 2: Experimental session

Participants reported to a temperature-controlled laboratory (23 ± 1°C) individually, for the experimental session. Participants were instructed to refrain from strenuous exercise, consuming caffeine, and alcohol for 12 hours and food was restricted for 2 hours prior to the experimental session. Upon arrival to the laboratory, a small sample of urine was collected from all female participants to confirm a negative pregnancy test. Participants self-reported sleep durations from the night prior to experimental session, with an average of 7.0 ± 0.5 h of sleep. Laboratory personnel confirmed practice sessions were completed and uploaded to the data server. Then baseline body weight and blood pressure were measured, and participants were instrumented for heart rate and S_p_O_2_. Participants then completed 5 repeats of a sensorimotor assessment over the course of 4 hours, each assessment consisting of 3 sets of the Adaptive Reflexshot task, with each set comprising 3 rounds with 3 different target sizes.

Following physiologic baseline measurements, participants entered the 2.8 m^2^ environmental chamber to complete a baseline assessment comprising 3 sets of the task in normoxia (F_i_O_2_ = 20.5 ± 0.2%). After completing the baseline assessment, participants sat in a chair in the environmental chamber and were allowed to watch a documentary of their choosing between assessments. The environmental chamber was then set to a hypoxic (F_i_O_2_ = 14%) or normoxic (F_i_O_2_ = 21%) environment, counterbalanced across participants. A F_i_O_2_ = 14% was chosen as the literature is unclear of the effects of hypoxia at this level and there is also operational relevance as underpressurized aircraft can be operated at this hypoxic exposure for extended periods of time [7]. Participants were blinded to order of exposure and asked via questionnaire to identify the exposure at 60 min and 90 min during each exposure. Participants completed an assessment (3 sets of the task) after 60 min of exposure to either hypoxia or normoxia and again after 90 min of continuous exposure, to see if longer exposure impacts cognitive performance. Following the 90 min assessment, participants were given a 5 min bathroom break and then reentered the chamber. The F_i_O_2_ in the environmental chamber was changed to the opposite environmental condition. Again, participants completed an assessment (3 sets of the task) after 60 min of exposure to the experimental conditions and again after 90 min of exposure. Data were uploaded to a server and participants were free to leave the lab after deinstrumentation.

### Data and statistical analysis

Prior to analyses, all data were checked for outliers and the necessary assumptions for the repeated measures ANOVA, Pearson’s Chi-square test, and linear mixed effects models, and no corrections were necessary. Comparisons of physiological variables (S_p_O_2_, HR) were assessed using two-way repeated measures ANOVA for condition (hypoxia and normoxia), time (60 and 90 min), and their interaction. If a significant main effect or interaction was observed, post-hoc tests using Sidak’s multiple comparison tests were completed. Pearson’s Chi-square test was used to assess subject blinding of environmental conditions.

Consistent with testing our hypothesis, a linear mixed effects model (lme4 and lmerTest R packages) was used to test for a change in motor acuity. The main outcome performance metrics were shot time (median elapsed time between target appearance and first shot, in units seconds), and shot variability (median angular distance between target center and the location of first shot, in units of degress). Changes in these performance metrics are reflective of changes in speed (1/(shot time)) or precision (1/(shot variability)), respectively. Other performance metrics measured, separately for each target size, during the gamified sensorimotor task included total number of shots taken (#), total number of targets (# of the number of targets spawned across rounds), hits (#, total number of shots that hit targets), misses (#, total number of missed shots), and hit rate (%, percentage of hit versus missed shots across rounds). The linear mixed effects models predictor variables included the baseline performance metric (performance metrics within each target size during baseline assessment), time (the start time of each round relative to the beginning of the experimental session), shot time for small targets (relative to shot time for large targets), shot time for medium targets (relative to shot time for large targets), shot variability for small targets (relative to shot variability for large targets), shot variability for medium targets (relative to shot variability for large targets), S_p_O_2_ decrease from baseline (the drop, compared to baseline performance trials, in arterial oxygen saturation), and the interactions of these variables. In these models, the time variable was transformed to log(time) because, based on large data sets from Aim Lab, player performance changes non-linearly over time within a day as well as across days and log(time) is a reasonable approximation of the relationship between time and performance change as performance approaches asymptote [9]. We utilized a type 3 sum of squares ANOVA table for significance of the individual decrease in S_p_O_2_ from baseline_,_ time, and their interaction on the gamified sensorimotor performance metric variables. Particular analyses of interest were the main effect of S_p_O_2_ decrease on performance metrics and the interactions between S_P_O_2_ decrease and other predictors on performance metrics. Data are presented as main and interaction effect coefficient estimates as a measure of the magnitude and direction of the effect on each performance measure and the associated p-value. All data were analyzed using R version 4.1.3 (R foundation for Statistical Computing). A priori statistical significance was set at *P* ≤ 0.05, and actual p-values are reported where possible. Group mean data are reported as mean ± standard deviation, and mean differences (diff.) and 95% confidence intervals (CI) are reported for multiple comparisons.

## Results

### Environmental and physiological variables

There was no difference in S_p_O_2_ between normoxia timepoints (S_p_O_2(60 min)_: 98 ± 2%; S_p_O_2(90 min)_: 99 ± 1%, mean diff. 0%, 95% CI -0.8, 1.1, p = 0.90), as well as no difference between hypoxic timepoints (S_p_O_2(60 min)_: 89 ± 3%; S_p_O_2(90 min)_: 90 ± 2%, mean diff. 1%, 95% CI—0.2, 1.7, p = 0.12). However, participants had lower S_p_O_2_ during both hypoxia timepoints compared to normoxia timepoints (p < 0.01) with a mean difference between conditions of -9% (95% CI -10, -8) at 60 mins and a mean difference of -9% (95% CI -10, -8) at 90 mins. Heart rate did not differ between normoxia timepoints (HR_(60 min)_: 74 ± 13 bpm, HR_(90 min)_: 71 ± 13 bpm, mean diff. 3, 95% CI -2, 9, p = 0.27), nor did heart rate differ between hypoxia timepoints (HR_(60 min)_: 75 ± 14 bpm, HR_(90 min)_: 75 ± 14 bpm, mean diff. 1, 95% CI -5, 6, p = 0.96). Heart rate was not different between the conditions at 60 min (mean diff. 1, 95% CI -4, 7, p = 0.86) or at 90 min during hypoxia compared to normoxia (mean diff. 4, 95% CI -1, 9, p = 0.18). While participants were not informed of the environmental conditions, they were able to guess which condition they were in as evidenced by a Pearson’s Chi-square test (p = 0.04). Participants correctly identified the exposure condition 71% of the time when looking at every timepoint combined. However, when looking at the hypoxia condition subjects correctly only identified the condition correctly 66% and 56% of the time for 60 min and 90 min of hypoxia exposure.

### Gamified sensorimotor task: Performance

#### Effects of baseline performance, time, and target size on performance metrics, independent of hypoxia

Tables 1 and 2 show that all performance metrics improved with increased baseline performance (p <0.01). Both shot time (p < 0.01) and shot variability (p = 0.02), depended on time, demonstrating improved performance throughout the experimental session, reducing shot time by -0.045 s and reducing shot variability by -0.044 degrees per subsequent assessment. The interaction of baseline performance and time showed a decrease in shot time (p < 0.03), shot variability (p < 0.01), and total number of shots (p < 0.03) for subsequent assessments, for a given level of baseline performance. There was no evidence for an interaction between baseline performance and time for the other performance metrics (p > 0.06). As target size was reduced from large to either medium or small there were decreases in shot variability (medium p < 0.01, small p <0.01), total number of shots (medium p < 0.01, small p <0.01), total number of targets (medium p < 0.01, small p <0.01), hits (medium p < 0.01, small p <0.01), and hit rate (medium p < 0.01, small p <0.01), while there was an increase in shot time (medium p < 0.01, small p <0.01) and no evidence for differences in misses (medium p = 0.76, small p = 0.90).

**Table 1:** Adaptive Reflexshot linear mixed effects model estimates for the motor acuity performance metrics.

	Shot time			Shot variability		
Predictors	Estimates	CI	p	Estimates	CI	p
(Intercept)	-0.444	-0.544 - -0.344	<0.001	0.645	0.537–0.753	<0.001
Baseline	0.599	0.521–0.677	<0.001	0.622	0.542–0.702	<0.001
Time	-0.045	-0.078 - -0.012	0.009	-0.044	-0.081–0.007	0.022
S_p_O_2_ Decrease	0.012	-0.006–0.030	0.194	0.023	0.003–0.043	0.024
Medium Targets	0.392	0.310–0.474	<0.001	-0.768	-0.899 – -0.637	<0.001
Small Targets	0.871	0.693–1.049	<0.001	-0.995	-1.166 – -0.824	<0.001
Baseline x S_p_O_2_ Decrease	0.006	-0.010–0.022	0.463	0.029	0.009–0.049	0.005
Baseline x Time	-0.034	-0.065 - -0.003	0.036	-0.065	-0.102 – -0.028	0.001
Random Effects						
σ^2^	0.03			0.04		
Τ_00_	0.02			0.01		
ICC	0.419			0.140		
*N*	30			30		
Observations	360			360		
Marginal R^2^ / conditional R^2^	0.947 / 0.969			0.959 / 0.965		

Model structures for each performance (outcome) metric with main and interaction effect coefficient estimates (Estimates) as a measure of their individual effect size, confidence intervals (CI), and associated p-values. Random effects show within-group variance (σ^2^), between group variance (Τ_00_) and intraclass-correlation coefficient (ICC). Significance was set at p < 0.05.

**Table 2:** Adaptive Reflexshot linear mixed effects model estimates for additional performance metrics.

	Total number of shots			Total number of targets			Hits			Misses			Hit rate		
Predictors	Estimates	CI	p	Estimates	CI	p	Estimates	CI	p	Estimates	CI	p	Estimates	CI	p
(Intercept)	0.150	0.019–0.281	0.030	0.086	-0.020–0.192	0.115	0.122	0.012–0.232	0.035	0.024	-0.158–0.206	0.798	0.138	-0.042–0.318	0.141
Baseline	0.740	0.660–0.820	<0.001	0.863	0.789–0.937	<0.001	0.801	0.723–0.879	<0.001	0.726	0.610–0.842	<0.001	0.672	0.554–0.790	<0.001
Time	-0.004	-0.047–0.039	0.844	0.011	-0.020–0.042	0.490	0.017	-0.014–0.048	0.297	-0.064	-0.156–0.028	0.182	0.069	-0.025–0.163	0.157
S_p_O_2_ Decrease	-0.024	-0.048- -0.001	0.045	-0.021	-0.039 - -0.003	0.014	-0.028	-0.046 - -0.010	0.001	-0.001	-0.050–0.049	0.976	-0.025	-0.076–0.026	0.327
Medium Targets	-0.161	-0.239–0.083	<0.001	-0.117	-0.186 - -0.048	0.001	-0.146	-0.217 - -0.075	<0.001	0.019	-0.103–0.141	0.757	-0.196	-0.319 - -0.073	0.002
Small Targets	-0.311	-0.431- -0.191	<0.001	-0.180	-0.296 - -0.064	0.003	-0.257	-0.375 - - 0.139	<0.001	-0.008	-0.133–0.117	0.901	-0.383	-0.516 - -0.250	<0.001
Baseline x S_p_O_2_ Decrease	0.007	-0.015 - 0.029	0.492	-0.004	-0.020–0.012	0.625	-0.003	-0.021–0.015	0.693	0.022	-0.023–0.067	0.344	0.10	-0.041–0.061	0.700
Baseline x Time	-0.048	-0.091–0.005	0.029	-0.032	-0.065–0.001	0.066	-0.028	-0.063–0.007	0.119	-0.062	-0.154–0.030	0.181	-0.079	-0.175–0.017	0.109
Random Effects															
σ^2^	0.05			0.02			0.03			0.22			0.23		
Τ_00_	0.09			0.05			0.06			0.15			0.15		
ICC	0.648			0.674			0.689			0.416			0.390		
*N*	30			30			30			30			30		
Observations	360			360			360			360			360		
Marginal R^2^ / conditional R^2^	0.835 / 0.942			0.917 / 0.973			0.902 / 0.969			0.568 / 0.747			0.542 / 0.721		

Model structures for each performance (outcome) metric with main and interaction effect coefficient estimates (Estimates) as a measure of their individual effect size, confidence intervals (CI), and associated p-values. Random effects show within-group variance (σ^2^), between group variance (Τ_00_) and intraclass-correlation coefficient (ICC). Significance was set at p < 0.05.

### Hypoxia’s effect on motor acuity

**Fig 4:**
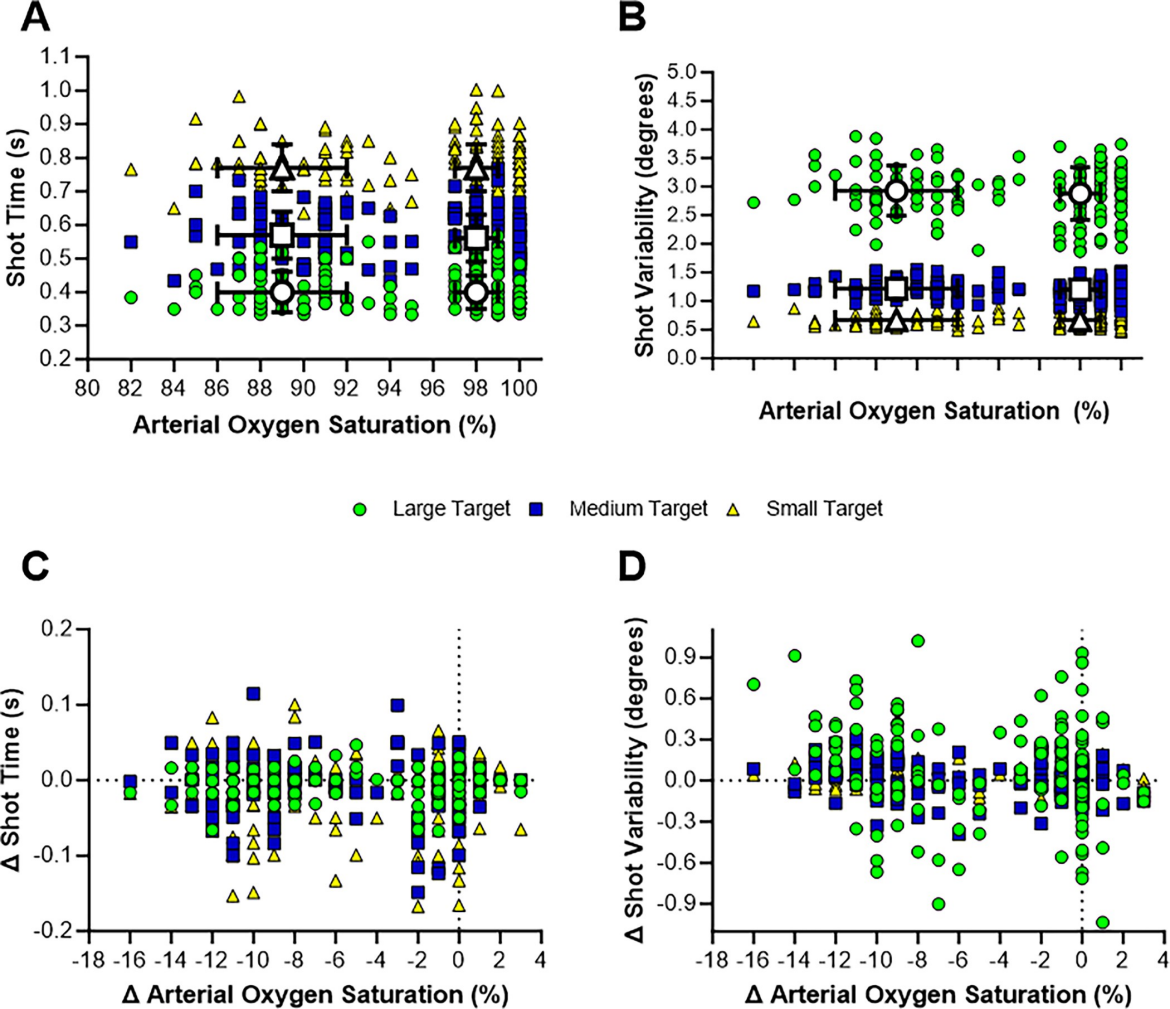
Visualization of motor acuity variables. Fig 4. Is a visual depiction of shot time (A) and shot variability (B) plotted over arterial oxygen saturations during each assessment and each target size. The figure shows the individual responses at each of the 5 assessment timepoints (i.e., baseline and the 60- and 90-min assessments during the normoxic and hypoxic conditions). Panels C and D depict the change (Δ) from baseline performance in shot time (C) and shot variability (D) plotted over the change in arterial oxygen saturations during each assessment and each target size. The green circles represent the large target size rounds. The blue squares represent the medium size target size rounds. The yellow triangles represent the small target size rounds. On panels A and B, the white circle, square, and triangle represent the average ± standard deviation (black bars) shot time or shot variability with the corresponding average ± standard deviation arterial oxygen saturation for the normoxic and hypoxic conditions. For statistical inferences of these data please see Tables 1 and 2.

Fig 4. shows shot time (A) and shot variability (B) plotted over the arterial oxygen saturation for each assessment and each target size within those assessment. Average shot time (collapsed across timepoint but not condition) increased from large targets (normoxia: 0.40 ± 0.05 s; hypoxia: 0.40 ± 0.06 s) to medium targets (normoxia: 0.56 ± 0.07 s; hypoxia: 0.57 ± 0.07 s) and to small targets (normoxia: 0.77 ± 0.07 s, p < 0.01; hypoxia: 0.77 ± 0.07 s, p < 0.01). Average shot variability (collapsed across timepoint but not condition) decreased from large targets (normoxia: 2.88 ± 0.046°; hypoxia: 2.93 ± 0.044°) to medium targets (1.20 ± 0.15°; hypoxia: 1.22 ± 0.15°) and to small targets (normoxia: 0.67 ± 0.11°, p < 0.01; hypoxia: 0.67 ± 0.10°, p <0.01). Table 1 shows results of linear mixed effects models for shot time and shot variability. There was no evidence that shot time was affected by decreases in S_p_O_2_ (p = 0.19) or the interaction of decreases in S_p_O_2_ and baseline (p = 0.46), which suggests no change in speed during moderate hypoxia exposure. However, shot variability increased by 0.023 degrees with decreases in S_p_O_2_ (p = 0.02). For a given baseline performance, shot variability increased by 0.029 degrees per each percent decrease in S_p_O_2_ (p < 0.01). Therefore, precision decreased with greater decreases in S_p_O_2._

### Hypoxia’s effect on the other performance metrics

Table 2 shows the results of linear effects mixed models for the other performance metrics. These models demonstrated a decrease in total numbers of shots (-0.024, p < 0.05), total number of targets (-0.021, p = 0.01), and the number of hits (-0.028, p < 0.01) with decreases in S_p_O_2,_ while there was no evidence for an effect of S_p_O_2_ decrease on misses (p = 0.98) or hit rate (p = 0.33)_._ The interaction of baseline performance and S_p_O_2_ decrease showed no evidence for an effect on any of the performance metrics (p > 0.34).

## Discussion

In support of our hypothesis, the gamified sensorimotor task (Adaptive Reflexshot) revealed alterations in motor acuity with hypoxia. Specifically, reductions in arterial oxygen saturation reduced precision without altering speed; greater shot variability with no evidence that shot time had changed. Decreasing precision without changing speed suggests that participants are not shifting along the same speed-precision tradeoff curve, but rather that participants are shifting to a different (lower performing) speed-precision tradeoff curve. Hence, motor acuity is impaired during moderate hypoxic exposure when individual differences in arterial oxygen saturation are considered.

The observed decrement in motor acuity when accounting for individual differences in S_p_O_2_ decreases demonstrates the individuality of hypoxic exposure responses and provides a foundation for future research in moderate hypoxia and cognition. Specifically, while much of the past research in moderate hypoxia and complex cognitive function has demonstrated mixed results with either no effect or negative effects (see reviews by Taylor et al. [2] and Virues-Ortega et al., [15], the current study utilized individual decrements in S_p_O_2_ instead of F_i_O_2_ to characterize the hypoxic condition. This characterization allows for the individualistic response to hypoxia to be accounted for when assessing cognitive performance. This is particularly important given the association between arterial oxygen saturation and cerebral oxygen saturation [16]. Moreover, this method of evaluating cognitive performance under hypoxia was a continuation of a secondary analysis from our prior study using a similar first person stylistic game (Gridshot, Statespace Labs, Inc.) [11]. In that study, we were underpowered to observe performance decrements using this statistical approach, although we did observe a ‘trend’ towards decreases in shot precision (p = 0.12) with decreasing arterial oxygen saturations [11], which was the impetus for the current study. Our previous and current studies align with literature showing that reductions in executive function are correlated with decreases in arterial oxygen desaturation. Specifically, Ochi et al. found a negative correlation (r = -0.293, p <0.01) of increases in Stroop Task Interference with decreased arterial oxygen saturation [17]. Interestingly, in the same study, there was not a clear delineation of increases in error rate or reaction time of the Stroop Task, when investigated by ANOVA, except at an F_i_O_2_ of 10.5% [17]. A more recent investigation by Williams et al. demonstrated that performance accuracy and reaction time of a complex central executive task (n-back) was reduced at F_i_O_2_ of 0.12 and that the reduction in performance correlated (accuracy r = 0.66 and reaction time = r = -0.36) with the change in S_p_O_2_ [18]_._ The current work, along with the previous investigations, demonstrate a connection between S_p_O_2_ and cognitive and sensory motor performance at moderate hypoxia, but also demonstrate that the model utilized for analysis is vital to interpretation, as the effects are subtle.

While the effects of moderate hypoxia were relatively small during this investigation, this study shows a proof of concept that highly engaging gamified cognitive assessments of sensorimotor performance are sensitive to detect performance decrements due to moderate hypoxic exposure, particularly when accounting for changes in arterial oxygen saturation. The subtle nature of these decrements in sensorimotor performance will likely affect tasks that require more precise movements, since only shot variability was affected (Table 1). Therefore, those personnel who perform job functions that require precise actions may be at greater risk of impaired performance. This may have implications for identifying those jobs at the highest risk of errors due to hypoxia exposure. Additionally, other factors may arise during hypoxic exposure and cause greater perturbations to cognition, via increased decrements in S_p_O_2_. Specifically, physically demanding and labor-intensive tasks would decrease arterial oxygen saturation of aircrews traveling in an unpressurized plane, which would increase the risk of poor sensorimotor task performance. Further research is needed to elucidate how specific job functions are affected at varying S_p_O_2_ levels.

### Experimental considerations

There are a couple of experimental considerations that must be acknowledged. First, the participants were exposed to normobaric hypoxia, which may physiologically differ from hypobaric hypoxia [5]. The differences may include greater hypoxemia, hypocapnia, and heart rate, but these differences between hypobaric and normobaric hypoxia tend to be relatively small [19]. Therefore, we think normobaric hypoxia was appropriate for this experimental design. Second, this investigation has not accounted for factors such as physical work, temperature, and humidity, which could also play an important role in determining the degree of impaired sensorimotor performance. Thus, this work is more applicable to pilots or personnel working in mostly stationary locations.

### Perspectives

Workers or military personnel exposed to low oxygen environments are at risk cognitive impairments, which may occur much earlier and in a more subtle manner than previous research has shown. Specifically, we demonstrate that sensorimotor performance is affected by moderate hypoxic conditions when considering the drop in S_P_O_2_. Because the observed changes in sensorimotor performance, and other domains of cognitive function demonstrated by other studies [17,18], were related to decrements in S_p_O_2_, this may provide the evidence needed to promote the monitoring of S_p_O_2_ as an easily assessed physiological biomarker to quantify the risk of declining function. Therefore, instead of focusing on F_i_O_2_ or the partial pressure of oxygen levels, future research should aim to identify the target levels of arterial oxygen saturation to best understand when and how hypoxia modifies cognitive function.

## Conclusion

Greater decreases in S_p_O_2_ during moderate hypoxic exposure can hinder sensorimotor task performance via decreases in precision with no change in speed. This suggests that participants who had greater S_p_O_2_ decreases would shift to a different, lower performing speed-precision tradeoff curve, rather than move to a different operating point on the same speed-precision curve. Thus, personnel who are exposed to moderate hypoxia and have greater decreases in S_p_O_2_ exhibit impaired motor acuity, i.e., less precise movements even though decision time and movement speed are unaffected.
